# Synthesis and in vitro α-glucosidase and cholinesterases inhibitory actions of water-soluble metallophthalocyanines bearing ({6-[3-(diethylamino)phenoxy]hexyl}oxy groups

**DOI:** 10.55730/1300-0527.3368

**Published:** 2022-01-18

**Authors:** Turgut KELEŞ, Zekeriya BIYIKLIOĞLU, Didem AKKAYA, Arzu ÖZEL, Burak BARUT

**Affiliations:** 1 Central Research Laboratory Application and Research Center, Recep Tayyip Erdoğan University, Rize, Turkey; 2 Department of Chemistry, Faculty of Science, Karadeniz Technical University, Trabzon, Turkey; 3 Department of Biochemistry, Faculty of Pharmacy, Karadeniz Technical University, Trabzon, Turkey

**Keywords:** Phthalocyanine, water-soluble, cholinesterases, α-Glucosidase, enzyme kinetic

## Abstract

In this paper, we have prepared peripherally tetra-({6-[3-(diethylamino)phenoxy]hexyl}oxy substituted cobalt(II), copper(II), manganese(III) phthalocyanines (**3, 4, 5)** and their water-soluble derivatives (**3a, 4a, 5a)**. Then, in vitro α-glucosidase and cholinesterases inhibitory actions of the water-soluble **3a, 4a, 5a** were examined using spectrophotometric methods. **4a** had the highest inhibitory effects among the tested compounds against α-glucosidase due to IC_50_ values. **4a** and **5a** had 40 fold higher inhibitory effects than the positive control. For cholinesterases, the compounds showed significant inhibitory actions that of galantamine which was used as a positive control. According to the SI value, **3a** inhibited acetylcholinesterase enzyme selectively. In kinetic studies, **4a** was a mixed inhibitor for α-glucosidase, **3a** was a competitive inhibitor for AChE, and **4a** was a mixed inhibitor for BuChE. The therapeutic potential of these compounds has been demonstrated by in vitro studies, but these data should be supported by further studies.

## 1. Introduction

Diabetes Mellitus (DM), which is a metabolic disease that occurs in advanced ages and is characterized by hyperglycemia, causes health complications such as retinopathy, nephropathy, cardiovascular disease, and obesity [1,2]. Controlling hyperglycemia and regulating blood sugar levels are the most important targets for this metabolic disease [3,4]. One of the most important approaches used in the treatment of DM is the inhibition of the α-glucosidase enzyme, which breaks down polysaccharides into smaller saccharides. With the inhibition of α-glucosidase, the hydrolysis of food-derived sugar is prevented, thereby controlling the blood sugar level and regulating postprandial hyperglycemia [5,6]. Although α-glucosidase inhibitors such as acarbose, miglitol, and voglibose are currently used in the treatment, various side effects such as nausea, diarrhea, and gastrointestinal discomfort are observed in the clinic [7,8]. The search for alternative α-glucosidase inhibitors to these drugs, which are less costly and have fewer side effects, continues [9,10].

Alzheimer’s disease (AD), is one of the neurodegenerative diseases that progresses over time and has limited treatment [11,12]. This disease causes memory, learning loss, and selective/irreversible impairments that occur in cholinergic function. This is called the “cholinergic hypothesis” in the pathogenesis of AD [13,14]. Acetylcholinesterase (AChE) and butyrylcholinesterase (BuChE) enzymes, which are responsible for the termination of the neurotransmission signal in the cholinergic hypothesis associated with learning and memory function, perform this task by hydrolyzing acetylcholine (ACh) to choline and acetic acid. Low ACh levels are a condition that should be treated in AD [15,16]. Today, cholinesterase (ChE) inhibitors such as galantamine, rivastigmine, donepezil, and tacrine are used for AD in clinic. However, its clinical usage has been limited due to various side effects such as hepatotoxicity, gastrointestinal system disorders, and hypotension arising [17,18].

Phthalocyanines have been used in various biological and industrial areas such as photodynamic therapy [19], anticancer agents [20], organic photovoltaic devices [21], liquid crystals [22], electrochromic display devices [23], solar cell [24], electrocatalytic agents [25] owing to their chemical and physical properties. Also, there are many studies on the α-glucosidase and cholinesterase inhibitory effects of phthalocyanines in the literature [26,27]. Diethylamino groups are widely preferred as substituted groups on water-soluble phthalocyanines, as they have both biological and pharmacological importance and contain nitrogen atoms that can be quaternized [28,29]. For this reason, the aim of this study is to determine the in vitro α-glucosidase and cholinesterases inhibitory effects of water-soluble cobalt(II), copper(II), manganese(III) phthalocyanines bearing peripherally tetra-({6-[3-(diethylamino)phenoxy]hexyl}oxy groups.

## 2. Experimental

The used materials, equipment, and in vitro inhibition assay on α-glucosidase, AChE, and BuChE are given in supplementary information.

### 2.1. Synthesis

#### 2.1.1. 4-({6-[3-(Diethylamino)phenoxy]hexyl}oxy)phthalonitrile (2)

6-[3-(Diethylamino)phenoxy]hexan-1-ol **(1)** (1 g, 3.78 mmol), 4-nitrophthalonitrile (0.65 g, 3.78 mmol), dry K_2_CO_3_ (1.6 g, 11.34 mmol) were dissolved in DMF (15 mL) at 60 °C under N_2_ atmosphere for 4 days. Then, reaction mixture was poured into water (200 mL) and stirred for 3 h. After that, the aqueous phase extracted with chloroform (3 × 50 mL). The combined extracts were dried over anhydrous MgSO_4_ and then filtered. The product was purified by aluminum oxide column chromatography using CHCl_3_. Yield: 0.5 g (34%). IR (ATR), ν/cm^−1^: 3075 (Ar–H), 2942–2870 (Aliph. C–H), 2230 (C ≡ N), 1598, 1501, 1473, 1394, 1315, 1281, 1255, 1213, 1169, 1096, 1022, 919, 803, 758. ^1^H NMR (400 MHz, DMSO-d*_6_*), (δ): 8.01 (d, 1H, Ar-H), 7.72 (s, 1H, Ar–H), 7.42 (d, 1H, Ar–H), 6.98 (t, 1H, Ar–H), 6.22 (d, 1H, Ar–H), 6.12–6.08 (m, 2H, Ar-H), 4.11 (t, 2H, CH_2_–O), 3.88 (t, 2H, CH_2_–O), 3.28–3.23 (q, 4H, –CH_2_-N), 1.73–1.66 (m, 4H, –CH_2_-), 1.45–1.39 (m, 4H, –CH_2_-), 1.05 (t, 6H, CH_3_). ^13^C-NMR (100 MHz, DMSO-*d_6_*), (δ): 162.48, 160.39, 149.14, 136.16, 130.19, 120.66, 120.46, 116.70, 116.68, 116.17, 106.16, 105.00, 101.20, 98.61, 69.41, 67.30, 44.09, 29.19, 28.61, 25.69, 25.52, 12.88. MS (ESI), (*m/z*) calcl. 391.50; found: 391.70 [M]^+^.

#### 2.1.2. The synthesis procedure of metallophthalocyanines (3, 4, 5)

4-({6-[3-(Diethylamino)phenoxy]hexyl}oxy)phthalonitrile **(2)** (100 mg, 0.25 mmol), 0.12 mmol related metal salts (16 mg CoCl_2_, 16 mg CuCl_2_, 17 mg MnCl_2_) in 1-pentanol (2 mL) and 1,8-diazabycyclo [5.4.0]undec-7-ene (DBU) (3 drops) was heated at 160 °C for 1 day. The product was precipitated with *n*-hexane. Finally, (**3, 4, 5**) were purified by aluminum oxide column chromatography using CHCl_3_.

#### 2.1.3. Cobalt(II) phthalocyanine (3)

Yield: 20 mg (20%), m.p. >250 °C. IR (ATR), ν/cm^−1^: 3066 (Ar–H), 2920–2850 (Aliph. C–H), 1729, 1607, 11497, 1488, 1467, 1357, 1232, 1214, 1178, 1094, 820, 748, 654. UV–Vis (DMF) λmax nm (log ɛ): 668 (4.93), 609 (4.51), 328 (4.92). MALDI-TOF-MS m/z calc. 1624.95; found: 1624.56 [M]^+^.

#### 2.1.4. Copper(II) phthalocyanine (4)

Yield: 26 mg (26%), m.p. > 250 °C. IR (ATR), ν/cm^−1^: 3071-3038 (Ar–H), 2968–2855 (Aliph. C–H), 1607, 1570, 1498, 1465, 1388, 1343, 1238, 1214, 1119, 1093, 956, 820, 745, 687. UV–Vis (DMF) λmax nm (log ɛ): 680 (5.02), 617 (4.679), 341 (4.86). MALDI-TOF-MS m/z calc. 1629.57; found: 1629.28 [M]^+^.

#### 2.1.5. Manganese(III) phthalocyanine chloride (5)

Yield: 50 mg (47%), m.p. > 250 °C. IR (ATR), ν/cm^−1^: 3071 (Ar–H), 2926–2858 (Aliph. C–H), 1603, 1496, 1464, 1341, 1240, 1214, 1123, 1074, 1052, 821, 745, 686. UV–Vis (DMF) λmax nm (log ɛ): 729 (4.98), 652 (4.59), 500 (4.36), 355 (4.85). MALDI-TOF-MS m/z calc. 1656.41; found: 1621.35 [M-Cl]^+^.

#### 2.1.6. The synthesis procedure of water-soluble metallophthalocyanines (3a, 4a, 5a)

**3a** (20 mg, 0.012 mmol), **4a** (20 mg, 0.016 mmol), **5a** (20 mg, 0.012 mmol) was dissolved in CHCl_3_ (2.5 mL), added iodomethane (2 mL) and stirred at rt for 4 days. The precipitated products were filtered and washed with CHCl_3_, diethyl ether.

#### 2.1.7. Water-soluble cobalt(II) phthalocyanine (3a)

Yield: 19 mg (70%), m.p. > 250 °C. IR (ATR), ν/cm^−1^: 3061 (Ar–H), 2932–2859 (Aliph. C–H), 1713, 1606, 1456, 1408, 1337, 1231, 1116, 1092, 1060, 1006, 870, 823, 750. UV-Vis (DMF), λ_maks_ (loge) nm : 671 (4.94), 608 (4.48), 327 (4.85). MALDI-TOF-MS m/z calc. 2192.71; found: 421.12 [M-4I]^+4^.

#### 2.1.8. Water-soluble copper(II) phthalocyanine (4a)

Yield: 20 mg (74%), m.p. > 250 °C. IR (ATR), ν/cm^−1^: 3013 (Ar–H), 2934–2860 (Aliph. C–H), 1607, 1487, 1457, 1390, 1232, 1090, 1053, 1005, 870, 745, 689. UV-Vis (DMF), λ_maks_ (loge) nm : 680 (5.03), 612 (4.44), 345 (4.70). MALDI-TOF-MS m/z calc. 2197.32; found: 422.24 [M-4I]^+4^.

#### 2.1.9. Water-soluble manganese(III) phthalocyanine chloride (5a)

Yield: 17 mg (65%), m.p. > 250 °C. IR (ATR), ν/cm^−1^: 3019 (Ar–H), 2933–2860 (Aliph. C–H), 1602, 1457, 1391, 1338, 1235, 1119, 1054, 993, 1054, 999, 869, 745, 691. UV-Vis (DMF), λ_maks_ (loge) nm : 728 (5.01), 661 (4.55), 500 (4.37), 367 (4.85). MALDI-TOF-MS m/z calc. 2224.17; found: 420.12 [M-Cl-4I]^+4^.

## 3. Results and discussion

### 3.1. Synthesis and characterization

**Figure 1:**
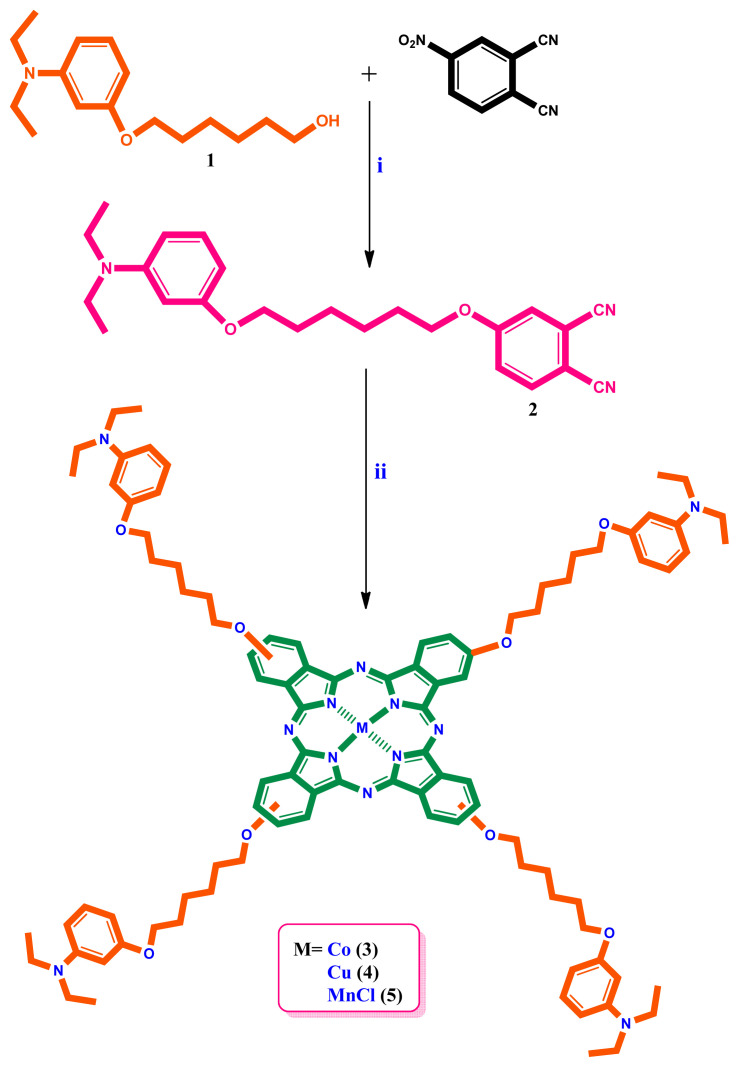
Synthesis of metallophthalocyanines. (i) K_2_CO_3_, 60 °C, DMF. (ii) CoCl_2_, CuCl_2_, MnCl_2_, 1-pentanol, DBU, 160 °C.

**Figure 2:**
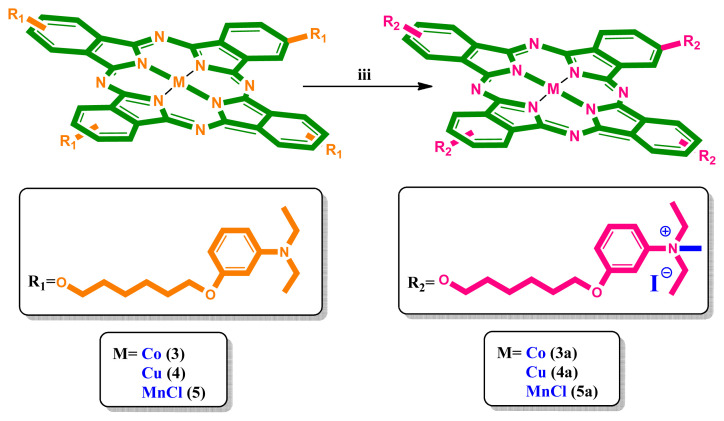
Synthesis of water-soluble phthalocyanines. (iii) CHCl_3_, CH_3_-I, rt.

**Figure 3:**
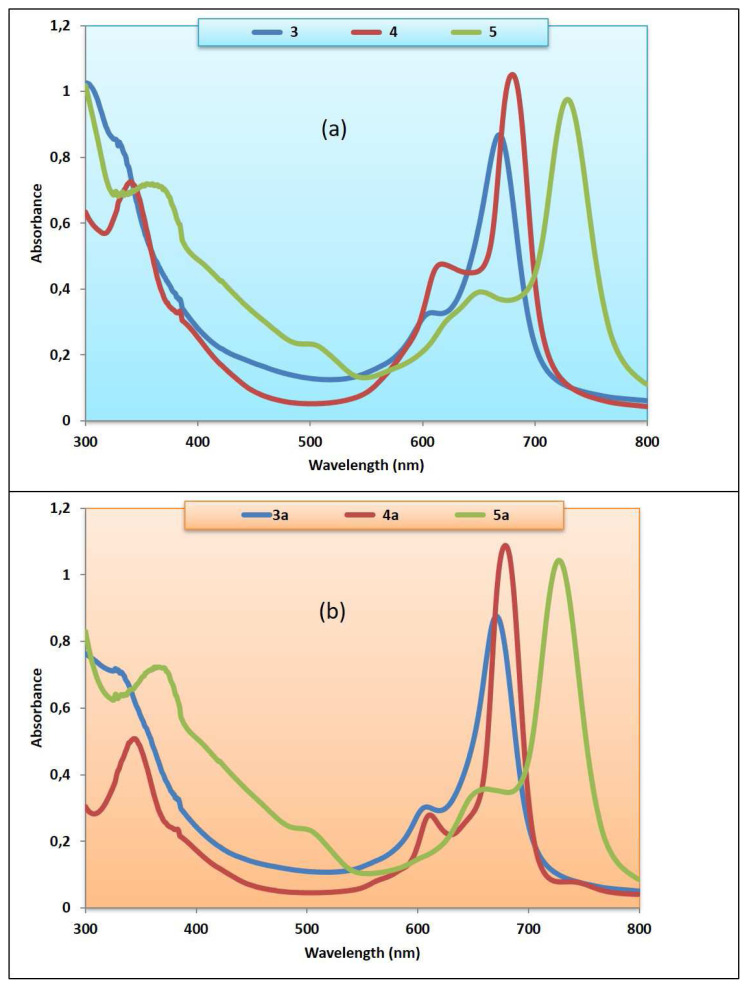
UV-vis spectra of **3, 4, 5** (a) and **3a, 4a, 5a** (b) in DMF.

The synthesis of peripherally tetra-({6-[3-(diethylamino)phenoxy]hexyl}oxy) group substituted metallophthalocyanines and their water-soluble derivatives are given in Figure 1 and 2. 4-({6-[3-(Diethylamino)phenoxy]hexyl}oxy)phthalonitrile (**2**) was prepared by the reaction of 6-[3-(diethylamino)phenoxy]hexan-1-ol (**1**) [30] and 4-nitrophthalonitrile in DMF at 60 °C. Cobalt(II), copper(II), and manganese(III) phthalocyanines (**3, 4, 5**) were synthesized by reaction of **2** and metal salts in 1-pentanol. Then, water-soluble phthalocyanines (**3a, 4a, 5a**) were prepared by reaction of cobalt(II), copper(II), and manganese(III) phthalocyanines (**3, 4, 5**) with iodomethane at room temperature [31,32]. In the IR spectrum of **2**, the new vibration belonging to C ≡ N group appeared as expected. In the ^1^H-NMR spectrum of **2**, aromatic protons were observed at *δ* 8.01–6.08 ppm. Also, CH_2_–O (4.11, 3.88 ppm), CH_2_-N (3.28-3.23), and –CH_3_ (1.05 ppm) protons were seen as expected. In the ^13^C-NMR spectrum of **2**, the nitrile group of carbon atom resonances was seen between 116.70 and 116.17 ppm. In the mass spectrum of 4-({6-[3-(diethylamino)phenoxy]hexyl}oxy)phthalonitrile (**2**), the [M]^+^ peak at 391.70 verified the suggested structure. In the IR spectra of (**3, 4, 5**), the sharp –C ≡ N vibrations of **2** disappeared as expected. The ^1^H-NMR spectra of (**3, 4, 5**) could not be recorded due to the paramagnetic metal ions in the ring center [33]. Also, the IR spectra of (**3a, 4a, 5a**) were very similar to (**3, 4, 5**). In the MALDI–TOF–MS of (**3, 4, 5**) the presence of the molecular ion peaks at m/z = 1624.56 [M]^+^, m/z = 1629.28 [M]^+^, m/z = 1621.35 [M-Cl]^+^ confirmed the proposed structure, respectively. On the other hand, the [(M-4I)/4]^+^ signals were observed at 421.12 [(M-4I)/4]^+^, 422.24 [(M-4I)/4]^+^ and 420.12 [(M-Cl-4I)/4]^+^ in mass spectra, supported the proposed structures of water-soluble (**3a, 4a, 5a**), respectively. The electronic absorption spectra of (**3, 4, 5**) and (**3a, 4a, 5a**) in DMF at room temperature are shown in Figure 3. As shown in Figure 3, the Q bands were observed as single and narrow bands at ~1×10^−5^ M. These bands confirm the monomeric behavior of the metallophthalocyanines (MPcs). The absorption spectra of (**3, 4, 5**) and (**3a, 4a, 5a**) displayed Q bands at 668, 680, 729, 671, 680, 728 nm, respectively. The B (Soret) bands of (**3, 4, 5**) and (**3a, 4a, 5a**) were observed at 328, 341, 355, 327, 345, 367 nm, respectively. All spectral data confirmed the suggested structure of cobalt(II), copper(II), and manganese(III) phthalocyanines (**3, 4, 5**) and their water-soluble derivatives (**3a, 4a, 5a**).

### 3.2. Inhibition study of α-glucosidase

In this study, in vitro anti-α-glucosidase effects the compounds **3a, 4a, 5a** were investigated by spectrophotometric methods. Acarbose was used as a positive control. The results were tabulated as IC_50_ values in Table 1. All of the compounds showed higher inhibitory effects on α-glucosidase than that of acarbose (IC_50_ = 60.28 ± 3.42 μM). **4a** has the best inhibitory actions with 1.36 ± 0.01 μM of IC_50_ value among the tested compounds. **4a** and **5a** had an inhibitory activity about 40 times higher inhibitory activity than that of acarbose. According to the literature, Güzel et al. investigated inhibitory effects of peripheral furan-2-ylmethoxy-substituted copper and manganese phthalocyanines on α-glucosidase [26]. The IC_50_ values of these compounds were determined as 911.20 μM and 695.37 μM, respectively. The peripherally tetra-({6-[3-(diethylamino)phenoxy]hexyl}oxy substituted cobalt(II), copper(II), manganese(III) phthalocyanines displayed a higher inhibitory effect on α-glucosidase than that of furan-2-ylmethoxy-substituted compounds on α-glucosidase, according to the IC_50_ values.

**Figure 4:**
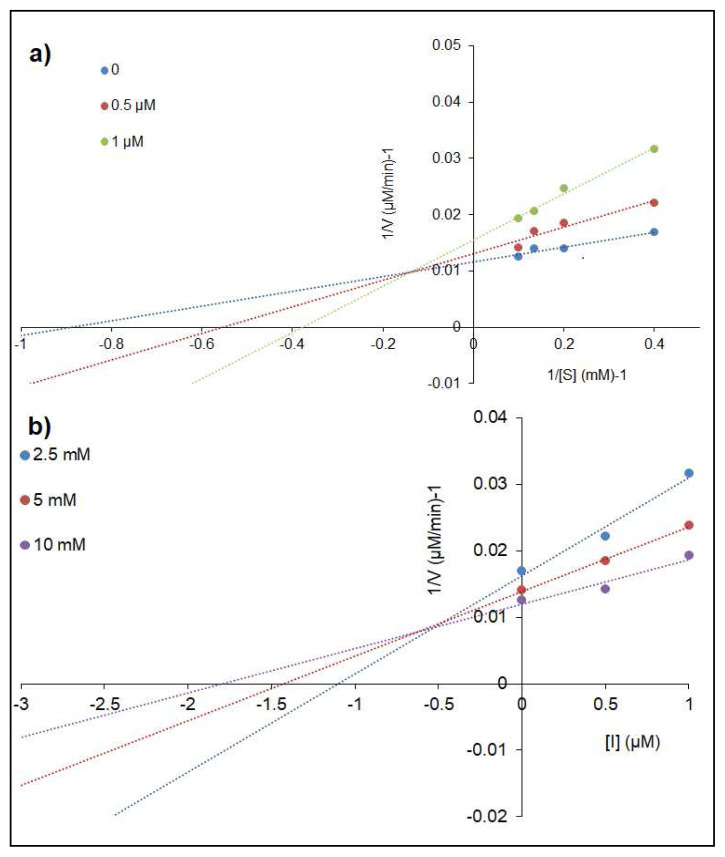
Lineweaver-Burk (a) and Dixon (b) plots of **4a** on α-glucosidase.

In this work, Lineweaver-Burk and Dixon plots were investigated to evaluate the inhibitory type and inhibition constant (*K_i_*) for **4a** which had the best inhibitory actions on α-glucosidase. The results are given in Table 2 and Figure 4. On enhancing substrate and inhibitors concentrations against α-glucosidase, *V_max_* (maximum rate) value diminished and *K_m_* value increased. This result claimed that the compound inhibited the enzyme via mixed inhibition.

### 3.3. Inhibition studies of AChE and BuChE

The in vitro antiChEs actions of all synthesized compounds were investigated to determine their therapeutic potential in AD. Galantamine was used as a positive control. The results are shown in Table 3. The results showed that the compounds have higher inhibition efficiency on AChE and BuChE when compared to galantamine (IC_50_ = 30.20 ± 0.58 μM for AChE and 52.04 ± 0.55 μM for BuChE). The IC_50_ values of **3a, 4a, 5a** were 0.65 ± 0.01 μM, 1.08 ± 0.03 μM, and 1.35 ± 0.01 μM, respectively on AChE. In addition, the compounds displayed significant BuChE inhibitory properties that of galantamine (p < 0.0001). **4a** had the best BuChE inhibitory actions with 0.29 ± 0.01 μM of IC_50_ value following **3a** with 3.06 ± 0.02 μM of IC_50_ value. According to the SI (selective index (IC_50_ = BuChE/IC_50_ = AChE)) values, **3a** inhibited AChE selectively (SI value = 4.70). The difference in the results is thought to be due to the metal effect. Frasco et al. reported the metals inhibitory effects of different metals (copper, nickel, zinc, cadmium, and mercury) on AChE [34]. The results showed that copper, zinc, cadmium, and mercury inhibited on AChE [34].

In literature, Arslan reported novel peripherally tetra-chalcone substituted metal-free, manganese, cobalt and copper phthalocyanines and their inhibitory effects against AChE [34]. These compounds had lower inhibitory effects than neostigmine (IC_50_ = 0.136 ± 0.011 μM) which was used as a positive control but **3a** showed higher inhibitory actions about 46 times than galantamine on AChE [35]. In our previous study, the ChEs inhibitory effects of peripheral or nonperipheral tetra-[4-(9H-carbazol-9-yl)phenoxy] substituted cobalt, manganese phthalocyanines were investigated and IC_50_ values of these compounds were determined ranging from 7.39 ± 0.25 μM to 58.02 ± 4.94 μM [36]. The compounds used in this study were found to be more effective when compared to our previous study according to the IC_50_ values [36].

**Figure 5:**
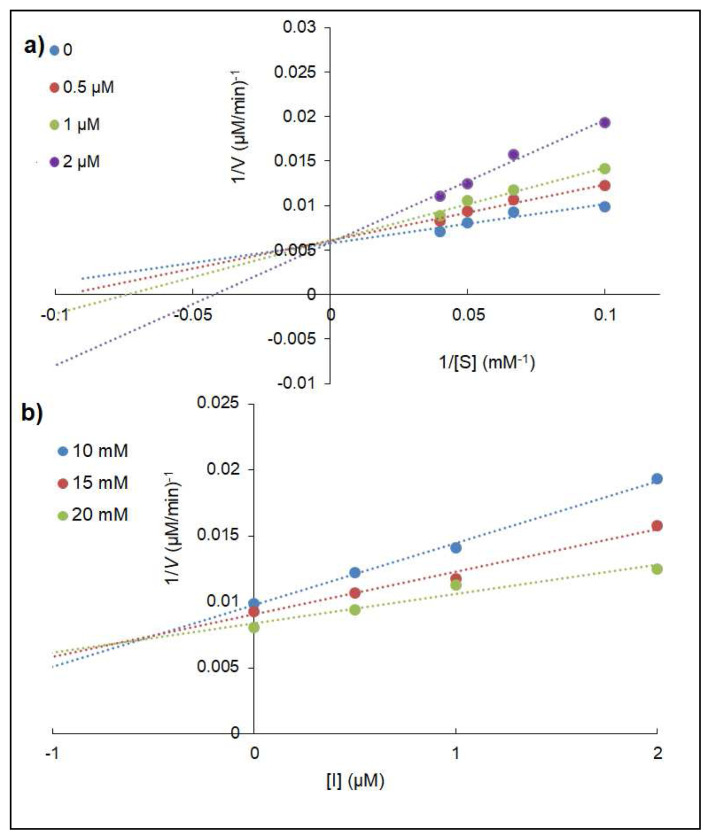
Lineweaver-Burk (a) and Dixon (b) plots of **3a** on AChE.

**Figure 6:**
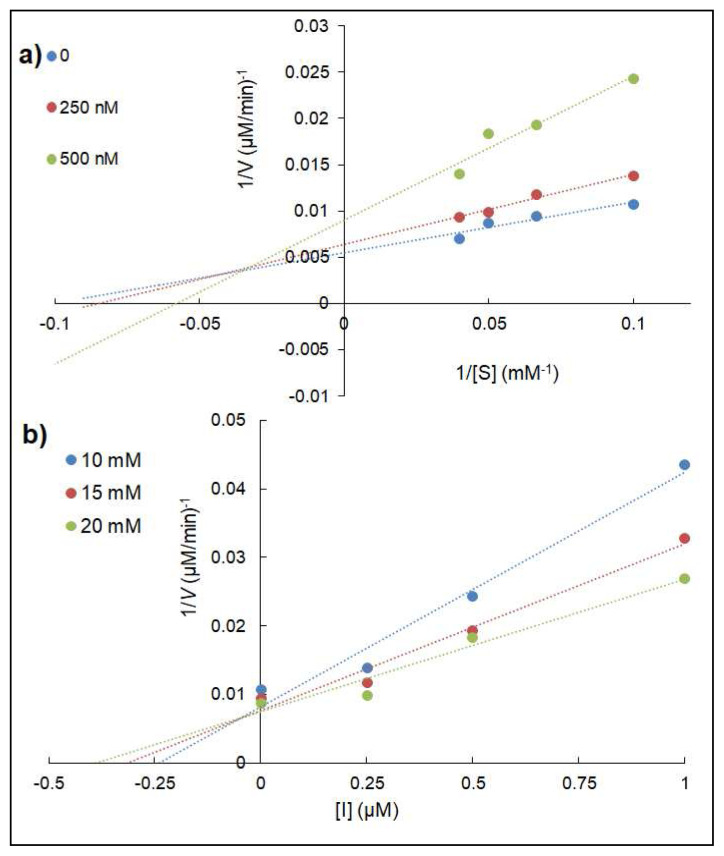
Lineweaver-Burk (a) and Dixon (b) plots of **4a** on BuChE.

In kinetic analysis, **3a** showed competitive inhibition with *V_max_* unchanged and *K_m_* increased on AChE, according to the Lineweaver-Burk plot (Figure 5a). On the other hand, **3a** was a mixed inhibitor due to the change of *V_max_* and *K_m_* values toward BuChE (Figure 6a). *K_i_* values of the compounds were determined as 0.51 ± 0.04 μM for **3a** and 0.05 ± 0.01 μM for **4a**, respectively (Figures 5b and 6b).

## 4. Conclusion

In this work, we have synthesized and characterized peripherally tetra-({6-[3-(diethylamino)phenoxy]hexyl}oxy) substituted metallophthalocyanines (**3, 4, 5)** and their water-soluble derivatives (**3a, 4a, 5a)**. Also, we have reported α-glucosidase and ChEs inhibitory actions of **3a, 4a, 5a** using spectrophotometric methods. All compounds had significant inhibitory properties on α-glucosidase and ChEs. According to the IC_50_ values, **4a** had the highest inhibitory effects among the tested compounds against α-glucosidase. **4a** and **5a** showed 40 fold higher inhibitory effects than acarbose. In ChEs studies, the compounds had significant inhibitory actions when compared to galantamine (p < 0.0001). **3a** inhibited the AChE enzyme selectively, according to the SI value. In kinetic studies, **4a** was a mixed inhibitor for α-glucosidase, **3a** was a competitive inhibitor for AChE, and **4a** was a mixed inhibitor for BuChE. Although it has been determined that these compounds have potential against DM and AD treatments in vitro, these data should be supported by further studies.

## Supplementary Information

### 1. Materials and methods

All reagents and solvents were of reagent grade quality and were obtained from commercial suppliers. Acarbose (Sigma, A8980), acetylcholinesterase (AChE) from *Electrophorus electricus* (electric eel) (Sigma, C3389), acetylthiocholine iodide (AChI) (Sigma, A5751), butyrylcholinesterase (BuChE) from equine serum (Sigma, C1057), butyrylthiocholine iodide (BChI) (Sigma, 20820), 5,5-dithio-bis(2-nitrobenzoic)acid (DTNB) (Sigma, D8130), galantamine (Sigma, 1287755), α-1,4-glucosidase from *Saccharomyces cerevisiae* (Sigma, G5003), and p-nitrophenyl-α-glucopyranoside (4-pNPG) (Sigma, 487506) were obtained from commercial sources. The inhibitory effects of the enzymes were performed using Thermo Scientific MultiskanTM Go Microplate Spectrophotometer using a 96-well microplate reader.

### 2. α-Glucosidase inhibition assay

α-Glucosidase inhibition assay was performed as previously reported with some modifications [1]. Acarbose was used as a positive control, and DMSO (final concentration .8%) as blank. The compounds (50 μL) in phosphate buffer pH 6.9, α-glucosidase (100 μL, 0.5 U/mL) were added and allowed to react for 20 min in a microplate. After incubation, 4-pNPG (50 μL, 5 mM) was added and incubated for 20 min at room temperature. The absorbance was measured at 405 nm using a 96-well microplate reader. α-glucosidase inhibition percentage of the compounds was calculated using formula 1.

### 3. In vitro AChE and BuChE inhibition assay

The in vitro AChE and BuChE inhibition actions were measured according to our previous method with some modifications [2]. Galantamine was used as a positive control and DMSO (final concentration 0.8%) as blank. Tris-HCl buffer (50 μL, 50 mM pH 8), DTNB (125 μL, 3 mM), AChE/BuChE (25 μL, 0.2 U/mL), and the compounds were added into the plate and incubated for 15 min at room temperature. Then, 25 μL of 15 mM of the substrate (AChI/BChI) was added into the plate to begin an enzymatic reaction. The absorbance was measured at 412 nm. AChE and BuChE inhibition percentage of the compounds was calculated using the formula. Formula = % Inhibition: ((A_control_-A)/A_control_) × 100. A_control_ is the activity of the enzyme without compound and A is the activity of the enzyme with compound. The inhibitory effect of the compounds was expressed as the concentration which inhibited 50% of the enzyme activity (IC_50_).

### 4. Kinetic analysis

Lineweaver-Burk and Dixon plots were performed to examine kinetic parameters (inhibitory type and constant (*K_i_*) values) of the compounds against enzymes [3,4]. The kinetic analysis was conducted by increasing substrate concentrations in the absence and presence of the compounds. The method was analyzed according to the procedures described above for α-glucosidase and cholinesterases.

### 5. Statistical analysis

Statistical analysis was performed using Microsoft Excel Windows 10 and GraphPad Prism 5.0. The results were expressed as mean ± standard deviation (n = 3). Statistical analysis was performed by one-way analysis of variance (ANOVA) followed by Tukey’s tests.

References1

ŞöhretoğluD
SariS
ÖzelA
BarutB

α-Glucosidase inhibitory effect of *Potentilla astracanica* and someisoflavones: Inhibition kinetics and mechanistic insights through in vitro and in silico studies
International Journal of Biological Macromolecules
2017
105
1062
1070
2875619710.1016/j.ijbiomac.2017.07.1322

BarutB
SariS
SabuncuoğluS
ÖzelA

Azole antifungal compounds could have dual cholinesterase inhibitory potential according to virtual screening, enzyme kinetics, and toxicity studies of an inhouse library
Journal of Molecular Structure
2021
1235
130268
3

LineweaverH
BurkD

The determination of enzyme dissociation constant
Journal ofAmerican Chemical Society
934
56
658
661
4

ButterworthP

The use of Dixon plots to study enzyme inhibition
Biochimica et Biophysica Acta (BBA) – Enzymology
1972
289
251
253
10.1016/0005-2744(72)90074-54631005

## Figures and Tables

**Table 1:** IC_50_ values of the compounds on α-glucosidase.

	α-Glucosidase
**3a**	2.48 ± 0.04***
**4a**	1.36 ± 0.01***
**5a**	1.49 ± 0.02***
**Acarbose**	60.28 ± 3.42

*** p < 0.0001 vs. positive control

**Table 2:** Kinetic parameters of the compounds on AChE, BuChE, and α-glucosidase.

	α-Glucosidase		AChE		BuChE	
	Type	*K* *_i_*	Type	*K* *_i_*	Type	*K* *_i_*
**3a**	-	-	competitive	0.51 ± 0.04	-	-
**4a**	mixed	0.49 ± 0.03	-	-	mixed	0.05 ± 0.01

**Table 3:** IC_50_ and SI values of the compounds on AChE, BuChE.

	AChE	BuChE	SI values (BuChE/AChE)
**3a**	0.65 ± 0.01***	3.06 ± 0.02***	4.70
**4a**	1.08 ± 0.03***	0.29 ± 0.01***	0.27
**5a**	1.35 ± 0.01***	3.57 ± 0.03***	2.42
**Galantamine**	30.20 ± 0.58	52.04 ± 0.55	1.72

*** p < 0.0001 vs. positive control
